# Novel design of tubular metamaterials with sign-switchable Poisson’s ratio and tunable mechanical properties for intestinal stents

**DOI:** 10.3389/fbioe.2026.1779512

**Published:** 2026-02-17

**Authors:** Yongtao Lyu, Volha Siniauskaya, Jian Jiang, Hao Wang, Lingqi Meng, Sergei Bosiakov, Mohammed Rafiq Abdul Kadir

**Affiliations:** 1 Department of Spinal Surgery, Central Hospital of Dalian University of Technology, Dalian University of Technology, Dalian, China; 2 Department of Engineering Mechanics, Dalian University of Technology, Dalian, China; 3 DUT-BSU Joint Institute, Dalian University of Technology, Dalian, China; 4 Faculty of Mechanics and Mathematics, Belarusian State University, Minsk, Belarus; 5 Department of Biomedical Engineering, Faculty of Engineering, Universiti Malaya, Kuala Lumpur, Malaysia

**Keywords:** adjustable energy absorption, intestinal stents, sign-switchable Poisson’s ratio, tubular mechanical metamaterial, tunable stiffness

## Abstract

**Introduction:**

Current intestinal stents used to restore patency face limitations due to the rigidity of metal structures and the premature degradation of biopolymer alternatives. Therefore, there is a critical need to develop stents that are flexible, radially strong, and able to adapt to the dynamic conditions within the body.

**Methods:**

This study introduces a novel tubular mechanical metamaterial featuring a sign-switchable Poisson’s ratio and tunable mechanical properties, achieved by integrating hexagonal unit cells with positive Poisson’s ratio and re-entrant unit cells with negative Poisson’s ratio. Experimental uniaxial compression tests and finite element analyses were performed to validate the proposed design and assess its mechanical performance.

**Results:**

The structure exhibits a negative Poisson’s ratio under tensile loading across all configurations, whereas under compression, the Poisson’s ratio was transited from negative to positive due to self-contact between triangular struts, enabling the distinctive sign-switching behavior. Experimental uniaxial compression tests and finite element analyses were performed to validate the proposed design and assess its mechanical performance. Results reveal that the geometric gap between the horizontal struts in the concave unit cells serves as a crucial tuning parameter: increasing this gap delays the onset of sign‐switching during compression while exerting minimal influence on the tensile response. The stiffness, yield strength, and energy absorption capacity are shown to be highly adjustable through this geometric control.

**Discussion:**

Overall, the metamaterial demonstrates superior energy absorption and tunable stiffness, making it a promising candidate for applications in intestinal stents.

## 1 Introduction

Intestinal obstruction is a life-threatening condition that requires urgent intervention. The main causes of obstruction include intra-abdominal adhesions, malignant tumours and hernias (Jeong and Park, 2020). Intestinal stents are medical implants designed to restore intestinal patency in cases of obstruction of various origins (Feng et al., 2022). However, despite considerable progress in intestinal stent technology, current options continue to exhibit significant limitations. Metal stents, which are the most widely used in clinical practice, possess high radial rigidity. Consequently, this stiffness can result in focal pressure on the bowel wall, representing a potential perforation risk (Chen et al., 2023; Ma et al., 2023). Despite the fact that biodegradable polymer stents resolve the issues of biodegradability and biocompatibility, they are not without limitations. For instance, there is a risk of early degradation, which could result in a loss of mechanical support (Fathi et al., 2020; Lin et al., 2023; Liu et al., 2025). Thermoplastic polyurethane (TPU) is considered a suitable alternative for intestinal stent applications, as it combines the requisite mechanical properties with biocompatibility (Wang et al., 2023). The TPU provides the required axial flexibility for implantation and radial stiffness to resist peristaltic compression (Hu et al., 2023; Soh et al., 2023).

Intestinal stents based on conventional materials, such as metals and biodegradable polymers, have shown considerable advances in recent years. However, these materials still fail to provide the optimal balance of mechanical properties required for the dynamic physiological environment, particularly the combination of flexibility, radial strength, and adaptability. This limitation has offered significant research interest in mechanical metamaterials. A critical design parameter for such advanced structures is their energy absorption capability, which is essential for mitigating the impact of cyclic peristaltic forces. This reduction in force transmission helps lower stress concentrations on the bowel wall and decreases the risk of stent migration.

Metamaterials are artificially engineered materials that exhibit extraordinary properties not typically observed in natural substances, primarily arising from their precisely designed microarchitectures (Kadic et al., 2013; Yasuda et al., 2020; Wu et al., 2021). These artificially engineered structures exhibit distinctive mechanical behaviors governed by both their material composition and the geometric configuration of their constituent elements. Notable metamaterials with a negative Poisson’s ratio (i.e., auxetics) are highly relevant for biomedical implants. Among these, auxetic tubular structures, a subclass of auxetic metamaterials, have garnered significant research attention due to their unique mechanical properties. In biomedical engineering, these structures have been employed in the design of vascular stents (Jiang et al., 2020; Abbaslou et al., 2023; Xiao et al., 2023; Ghofrani et al., 2024; Vellaparambil et al., 2024), bone screws (Yao et al., 2021; Barnett et al., 2024), etc*.*, owing to their capability to distribute loads uniformly and maintain structural integrity under large deformations.

A particularly innovative area of research focuses on the development of metamaterials exhibiting a sign-switchable Poisson’s ratio. Recent studies have explored hybrid structures that integrate regions with both positive Poisson’s ratio (PPR) and negative Poisson’s ratio (NPR) characteristics, thereby enabling precise tailoring of mechanical responses to meet specific functional requirements.

Most existing studies attribute the variation in Poisson’s ratio primarily to structural interactions that occur under different loading conditions (Chen et al., 2021; Lim, 2024; Wu et al., 2024). However, recent advancements have broadened this understanding. For instance, Park et al. (2018) demonstrated that the Poisson’s ratio can be reversibly tuned by integrating a thermosensitive composite with a high coefficient of thermal expansion and by exploiting the geometric nonlinearity of the structure. This combination enables a thermally induced transition between positive and negative Poisson’s ratio states, thereby enhancing the functional adaptability of the material under varying thermal environments. Building upon this principle of tunability, several approaches have been proposed to achieve sign-switchable Poisson’s ratio behavior. Lv et al. (2023) introduced a metamaterial design that utilizes self-contact mechanisms to mechanically regulate the Poisson’s ratio during deformation. Montazeri et al. (2023) developed a mechanically reconfigurable metamaterial in which both the sign of the Poisson’s ratio and the effective stiffness can be controlled by adjusting the geometric parameters of the unit cell. Likewise, Lyu et al. (2024) proposed a structure capable of independently and continuously tuning both the Poisson’s ratio and stiffness through precise geometric adjustments in response to different loading scenarios. Collectively, these studies highlight the increasing research emphasis on adaptive mechanical metamaterials, where the interplay between structural architecture and material composition enables programmable mechanical responses.

Although extensive research has been conducted on two-dimensional (2D) metamaterials exhibiting switchable Poisson’s ratios and tunable stiffness under various loading conditions, the extension of these capabilities to three-dimensional (3D) architectures remains relatively underexplored. For intestinal stents, such functionality is critical. Peristaltic motion requires geometric adjustment through sign-switchable auxeticity to prevent migration. However, the physiological loads require tunable stiffness to achieve a balance between luminal support and tissue damage. Additionally, adjustable energy absorption is required to mitigate risks from sudden impacts. Consequently, these adaptive properties can be integrated in order to develop improved stents that offer enhanced safety and performance.

In this study, we developed a novel tubular mechanical metamaterial by integrating a re-entrant structure exhibiting a negative Poisson’s ratio with a hexagonal unit cell characterized by a positive Poisson’s ratio. The introduction of self-contact within the design enables the emergence of unique mechanical properties, including a sign-switchable Poisson’s ratio, tunable stiffness, and adjustable energy absorption. The mechanical behavior of the proposed metamaterial was evaluated through a combination of experimental axial compression tests and finite element (FE) analyses. The validated FE model was subsequently employed to conduct a parametric study investigating the influence of geometric gap size on the structural response. The results demonstrate that the proposed metamaterial holds great potential for applications in intestinal stents.

## 2 Materials and methods

### 2.1 Structure design

**FIGURE 1:**
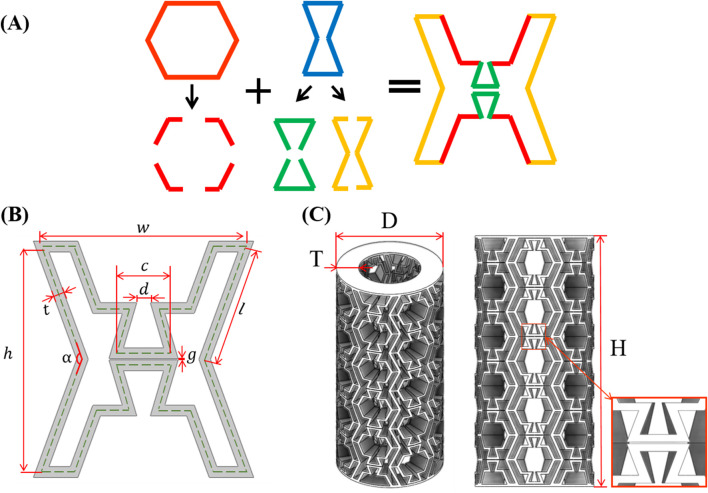
Design of the novel tubular metamaterial. **(A)** Design process of the unit cell. **(B)** Design parameters of the unit cell. **(C)** Overall view of the tubular structure.

The novel metamaterial developed in this study is based on a hybrid design that integrates hexagonal (convex) unit cells exhibiting a positive Poisson’s ratio with re-entrant (concave) unit cells characterized by a negative Poisson’s ratio. The convex unit cell is trimmed along both the vertical and horizontal directions, while the concave unit cell undergoes the removal of its upper and lower edges. Triangular supports derived from the re-entrant cell are incorporated into the geometry to enhance mechanical stability. The formation process of the unit cell for the proposed structure is illustrated in Figure 1A and the geometric parameters are defined in Figure 1B. In the proposed configuration, the width of the convex unit cell is denoted by w , the height of the concave unit cell by h , the length of the inclined struts by l , and the angle between the struts by α . The length of the horizontal struts in the concave unit cell is represented by c , while the vertical gap between these horizontal struts is denoted by g . The trimmed length of the hexagonal unit cell is represented by d , and the in-plane thickness of the struts by t . To enhance the generality of the parametric analysis and facilitate comparison across different scales, the geometric gap g is normalized by the in-plane thickness of the struts by t , thereby defining the dimensionless parameter $\bar{g}=g/t$ . The tubular lattice structure is then formed by periodically repeating and packing the unit cell along the cylindrical axis. In this configuration, the total height $\left(H\right)$ of the structure is 105.37 mm, and the total diameter $\left(D\right)$ is 47.94 mm. The out-of-plane thickness of each strut is denoted by T , as illustrated in Figure 1C.

### 2.2 Experimental approach

#### 2.2.1 Fabrication of specimens

The novel tubular metamaterials were fabricated using selective laser sintering (SLS) with an S-TPU 500 3D printer (Suzhou, China). Prior to fabrication, a computer-aided design (CAD) model was created using SolidWorks software (v2023, SolidWorks Inc., Massachusetts, USA). The resulting geometry was exported in STEP format and subsequently imported into the printer’s control system for processing. The specimens were printed with a nozzle tip diameter of 0.2 mm, a print speed of 30 mm/s and a layer height of 0.15 mm. Thermoplastic polyurethane (TPU) with a Shore hardness of 95 A, supplied by SUNLU (Zhuhai, Guangdong Province, China), was used as the base material. The geometric parameters of the fabricated 3D-printed structures are summarized in Table 1.

**TABLE 1:** The design parameters of the unit cells and the structure.

Parameter (unit)	w (mm)	h (mm)	l (mm)	c (mm)	α ( ° )	d (mm)	t (mm)	g (mm)
Value	18.66	20.67	11	4.99	140	1.22	1	0.05

#### 2.2.2 Determination of material property

**FIGURE 2:**
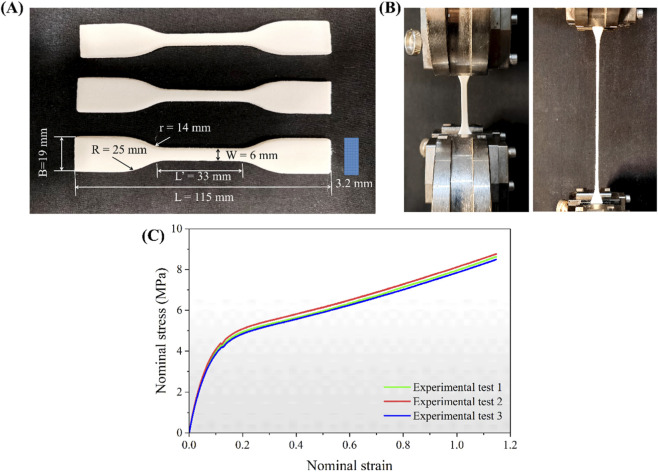
**(A)** Dumbbell-shaped TPU specimens and dimensions. **(B)** Tensile testing setup. **(C)** Experimental and fitted nominal stress-nominal strain curves for TPU.

The primary material properties were determined through uniaxial tensile tests. A standard dumbbell-shaped TPU specimen was modeled in accordance with the ASTM D638 international standard and fabricates as described in the previous section. The design and dimensions of the dumbbell specimen are shown in Figure 2A. As illustrated in Figure 2B, uniaxial tensile tests were performed on three specimens using an MTS universal testing machine (Model: MTS Criterion 43.104) equipped with a 10 kN load cell, which had a maximum measurement deviation of less than 1% of the indicated force. The tests were conducted at a constant crosshead speed of 10.0 mm/min, while load and displacement data were continuously recorded. The samples were held using self-locking wedge clamps, which ensured reliable fixation through the wedging action induced by the applied tensile load. The nominal stress-strain curves were derived from the measured load-displacement data and the initial specimen geometry. The nominal stress-strain curves were derived from the measured load-displacement data and the initial specimen geometry. A hyperelastic material constitutive model was then fitted to the experimental results. Several hyperelastic models–namely, Mooney-Rivlin, Polynomial, Neo-Hookean, Arruda-Boyce, and Yeoh–were compared with the experimental data. Among these, the Polynomial model (N = 2) provided the best fit to the experimental stress-strain curve, as shown in Figure 2C. Its strain energy density function is presented in Equation 1:

$$\begin{array}{c} W=C_{10}\left({\bar{I}}_{1}-3\right)+C_{01}\left({\bar{I}}_{2}-3\right)+C_{20}{\left({\bar{I}}_{1}-3\right)}^{2}+C_{11}\left({\bar{I}}_{1}-3\right)\left({\bar{I}}_{2}-3\right)\\ +C_{02}{\left({\bar{I}}_{2}-3\right)}^{2}+\frac{1}{D_{1}}{\left(J-1\right)}^{2}+\frac{1}{D_{2}}{\left(J-1\right)}^{4} \end{array}$$ (1)

where ${\bar{I}}_{1}$ and ${\bar{I}}_{2}$ are the strain invariants, J determinant of the deformation gradient, and $C_{10}$ , $C_{01}$ , $C_{11}$ , $C_{20}$ , $C_{02}$ , $D_{1}$ , and $D_{2}$ are the hyperelastic constants. The identified material parameters from the Polynomial (N = 2) model were subsequently implemented in Abaqus (v2021, Dassault Systèmes SIMULIA, USA) to define the TPU material behavior. The resulting parameters are summarized in Table 2.

**TABLE 2:** Material parameters obtained from quasi-static testing on TPU specimens.

ρ $\left(g/{cm}^{3}\right)$	$C_{10}$	$C_{01}$	$C_{20}$	$C_{02}$	$C_{11}$	$D_{1}$	$D_{2}$
1.15	−9.81	17.64	−5.89E-03	2.8	8.85E-02	0	0

#### 2.2.3 Quasi-static loading tests

**FIGURE 3:**
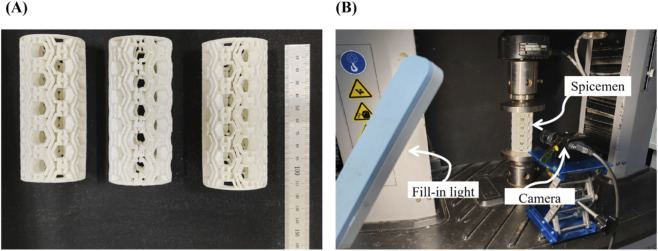
3D-printed structure and uniaxial quasi-static compression loading configuration. **(A)** 3D-printed samples. **(B)** Experimental setup for uniaxial compression testing.

The quasi-static mechanical behavior of the proposed structure was evaluated under compressive loading. During compression, it was observed that the exposed structural walls could come into direct contact with the pressure plates, and their bending deformation might induce premature structural failure. To mitigate this effect, 1.0 mm thick plates made of the same material were attached to the top and bottom surfaces of the specimen to ensure uniform load distribution and reduce stress concentrations during testing, as illustrated in Figure 3A. The printed specimens had an out-of-plane thickness of 10.0 mm, which was sufficient to prevent out-of-plane instability under the applied compressive loads.

Compressive loads were applied to the specimens at a constant rate of 2.0 mm/min, and the corresponding load-displacement data were recorded. In the test setup, two flat platens were positioned above and below the specimens, with the specimens centered on the platform. The force and displacement sensors were connected to the upper and lower platens to collect the reaction forces and displacement data from the experiment during the compression process. The tests were conducted using an MTS universal testing machine equipped with a 10.0 kN load cell and a maximum force measurement error of less than ±1%. Each specimen was compressed to a displacement of 52.0 mm, corresponding to an engineering strain of 0.5. A high-resolution camera was positioned in front of the experimental setup to capture the deformation of the structures throughout the testing process. The recorded images were subsequently analyzed to derive the compressive stress-strain curves.

Details regarding the calculation of stress-strain response, energy absorption, and specific energy absorption are provided in the following section. The quasi-static uniaxial compression test setup is shown in Figure 3B.

#### 2.2.4 Identification of parameters in the constitutive model

**FIGURE 4:**
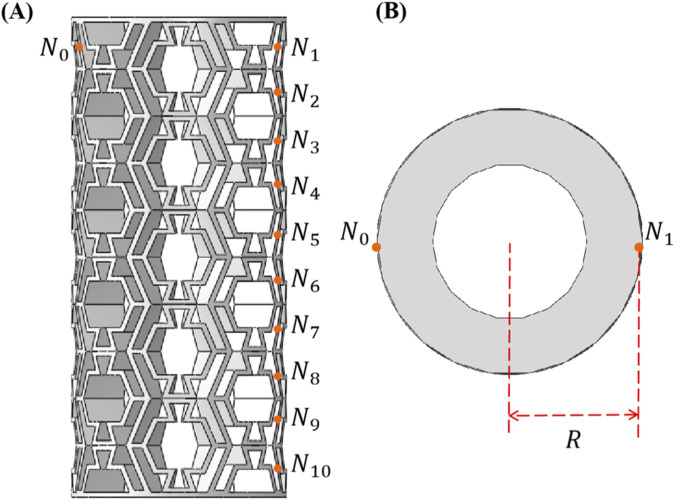
Calculation of effective Poisson’s ratio in two directions: **(A)** Front view. **(B)** Top view.

The mechanical performance of the tubular metamaterials was characterized using several key parameters, among which the effective Poisson’s ratio is particularly important, as it quantifies the material’s transverse strain response under axial loading. To evaluate variations in the global Poisson’s ratio of the structures, eleven markers were strategically placed on each experimental specimen and its corresponding FE model, as illustrated in Figure 4. Given the non-uniform distribution of orthogonal deformation across the specimen surface, the local Poisson’s ratio at each point was calculated, as shown in Equation 2:

νi=−ΔxiRΔy0H 1≤i≤10 (2)

The average Poisson’s ratio was determined by calculating the mean value of the effective Poisson’s ratio obtained from measurements at these characteristic points is expressed in Equation 3:

$$\bar{\nu }=\frac{1}{10}\sum_{1}^{10}\nu_{i}\left(1\leq i\leq 10\right)$$ (3)

where ${\Delta x}_{i}$ denotes the deformation of point $N_{i}$ in the diametral direction, and ${\Delta y}_{0}$ represents the displacement of point $N_{0}$ along the height direction.

The tunable stiffness introduced in this study was characterized by its dependence on deformation and was quantitatively evaluated using the effective Young’s modulus. The effective Young’s modulus ( E ) was defined as the ratio of the nominal stress applied to the structure to the corresponding nominal axial strain of the tube, measured within the initial linear portion of the stress-strain curve. This parameter was calculated using the following Equation 4:

E=FAΔhH (4)

where F denotes the reaction force exerted by the structure under compressive displacement along the *y*-axis, *A* represents the cross-sectional area of the tube, ​ Δh refers to the displacement of the upper boundary of the structure in the *y*-direction, and *H* is the initial height of the tube.

During the initial loading phase, the tubular metamaterial exhibited elastic behavior characterized by a linear relationship between the applied stress and strain. The slope of the stress-strain curve in this region corresponded to the elastic modulus, a fundamental parameter that reflects the stiffness of the tubular metamaterial.

Once the yield limit was reached, localized plastic deformation initiated within the metamaterial structure. This marked the onset of the stress plateau region, characterized by a stress level that remains approximately constant or increases gradually with further strain. The average stress within the plateau region, denoted as $\sigma_{pl}$ , was calculated as shown in Equation 5:

$$\sigma_{pl}=\frac{\int_{\varepsilon_{y}}^{\varepsilon_{sd}}\sigma \left(\varepsilon \right)d\varepsilon }{\varepsilon_{sd}-\varepsilon_{y}}$$ (5)

where $\varepsilon_{y}$ and $\varepsilon_{sd}$ represent the yield strain and strain at densification, respectively.

The plateau region plays a critical role in energy absorption, as it allows substantial deformation to occur at an approximately constant stress level. This stage represents the primary phase of energy dissipation within the metamaterial. The effective compression stroke for energy absorption extends from the onset of external loading to the point of full densification. Beyond this densification threshold, additional strain leads to intense compaction of the structural elements, resulting in a sharp rise in stress. In this densification regime, the stress-strain curve exhibits a pronounced steepening, reflecting the transition of the metamaterial toward behavior characteristic of a solid body. This stage typically corresponds to the maximum energy absorption capacity of the structure.

The specific energy absorption (SEA) capacity is one of the most important criteria for evaluating the performance of metamaterials. The energy absorption (EA) was calculated as the area under the stress-strain curve up to a specified strain, as expressed by the following Equation 6:

$$EA=\int_{0}^{\varepsilon_{sd}}\sigma \left(\varepsilon \right)d\varepsilon$$ (6)

where $\varepsilon_{sd}$ is the strain at densification, and $\sigma \left(\varepsilon \right)$ corresponds to the stress associated with a given strain.

The specific energy absorption (SEA) was defined as the absorbed energy per unit mass and is given by Equation 7:

$$SEA=\frac{EA}{\rho_{m}\rho_{v}}$$ (7)

where $\rho_{m}$ denotes the density of the base material, and $\rho_{v}$ represents the relative density of the undeformed structure.

The energy absorption efficiency (EAE) was evaluated based on the extent to which the energy absorption potential is utilized at different stages of deformation, and is defined as shown in Equation 8:

$$\eta \left(\varepsilon \right)=\frac{\int_{0}^{\varepsilon }\sigma \left(\varepsilon \right)d\varepsilon }{\sigma \left(\varepsilon \right)}$$ (8)

The maximum efficiency value was achieved under the condition from Equation 9:

$${\left.\frac{d\eta \left(\varepsilon \right)}{d\varepsilon }\right|}_{\varepsilon =\varepsilon_{sd}}=0$$ (9)

Accordingly, this analysis enabled a comprehensive characterization of the mechanical behavior of the tubular metamaterials under loading, encompassing both elastic and plastic deformation stages as well as the overall energy absorption processes.

### 2.3 Finite element modeling

**FIGURE 5:**
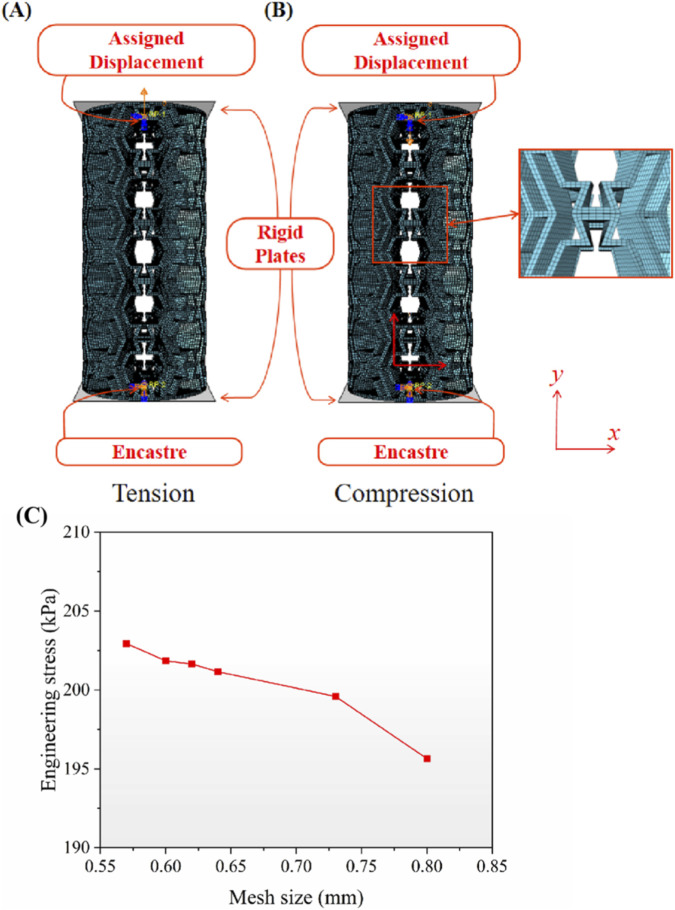
Boundary conditions and mesh configuration in the finite element models. **(A)** Uniaxial tension scenario. **(B)** Uniaxial compression scenario. **(C)** Mesh sensitivity analysis.

The finite element analysis (FEA) in this study was performed using the commercial software package Abaqus/Explicit 2021 (v2021, Dassault Systèmes SIMULIA, USA). The CAD models prepared in SolidWorks 2023 were imported into Abaqus, and the Polynomial (N = 2) hyperelastic model was employed to define the material properties of the structures. In the finite element model, the longitudinal direction in the plane was defined as the *y*-direction, and the transverse direction as the *x*-direction. As illustrated in Figures 5A,B, the structure was positioned between two rigid plates; a prescribed displacement boundary condition was applied to the reference point of the upper plate, while the lower plate was fully constrained using an encastre boundary condition. The structure was discretized using C3D8I (8-node linear brick, incompatible mode) elements with an average mesh seed size of 0.64 mm. A mesh sensitivity analysis confirmed that the results obtained with this mesh size were mesh-independent, as shown in Figure 5C. Furthermore, general contact was defined with a “Hard contact” formulation for the normal behavior and a “Penalty” formulation for tangential behavior, with a friction coefficient of 0.4.

## 3 Result and discussion

### 3.1 Comparison of compressive behaviors between FE and experimental results

**FIGURE 6:**
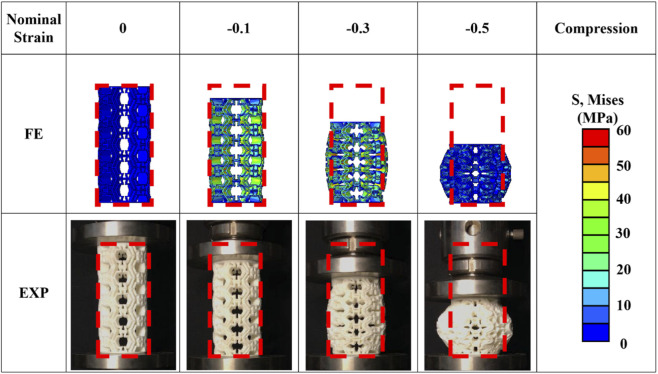
Comparison of the results between finite element analyses and experimental tests.

To validate the FE model of the tubular metamaterial under quasi-static uniaxial compression, experimental tests were conducted and compared with the FE results. Axial loading was simulated by applying a vertical displacement to the upper plate, with the nominal compressive strain reaching 0.5. The deformation process observed in both the experimental tests and FE simulations exhibited a high degree of consistency, as shown in Figure 6.

**FIGURE 7:**
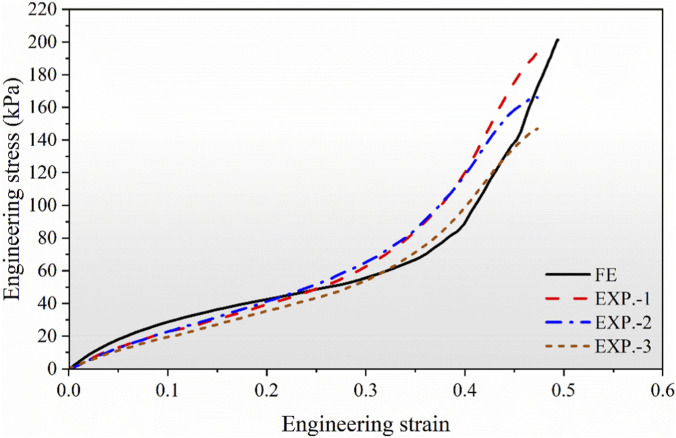
Relationship between engineering stress and strain of the proposed structure derived from experimental tests and finite element analyses.

The stress-strain curves shown in Figure 7 include the results from three experimental samples (EXP.-1, EXP.-2, EXP.-3) along with the FE simulation. At the initial loading stage, the experimental and numerical curves exhibit a close agreement, confirming the accuracy of the selected material parameters and boundary conditions. However, at higher strain levels (ε > 0.4), noticeable deviations appear, particularly in EXP.-3. These discrepancies are primarily attributed to imperfections in the 3D-printed specimens, such as wall thinning, non-uniform strut thickness, and deviations from the designed geometry. Because such manufacturing imperfections are not considered in the idealized FE model, the experimental samples showed slightly lower stiffness and load-bearing capacity compared with the FE results.

**FIGURE 8:**
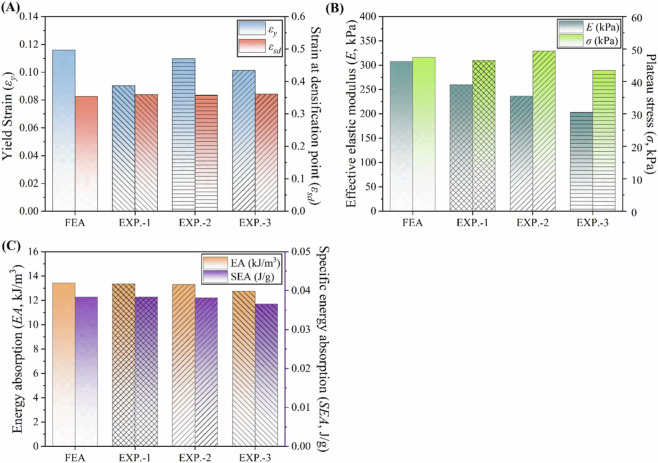
Comparison between FEA and experimental results. **(A)** Yield strain and strain at densification point. **(B)** Effective elastic modulus and plateau stress. **(C)** Energy absorption (EA) and specific energy absorption (SEA).

Key mechanical properties, including effective elastic modulus, yield strain, densification strain, and plateau stress, are illustrated in Figures 8A,B. The experimentally measured elastic modulus was approximately 27% lower than that from the FE prediction, likely due to geometric inaccuracies in the fabricated specimens. To reduce this discrepancy, future studies will employ additive manufacturing methods with higher resolution, which are capable of more accurately reproducing the designed geometry. To enable more rigorous correlation, subsequent work will focus on refining the numerical model to incorporate process-induced factors, including porosity and residual stresses. Furthermore, efforts will encompass sensitivity analyses of key parameters, along with the establishment of robust controls over the quality of feedstock and the input of energy with a view to enhancing material homogeneity and mitigating experimental variability. Nevertheless, the close agreement in yield strength and overall compressive behavior confirms the reliability of the FE model in accurately capturing the metamaterial’s structural response.

The current mechanical characterization was performed under quasi-static loading conditions. While this approach allows for a detailed analysis of the structure’s deformation mechanisms and energy absorption capacity, it does not capture the dynamic, cyclic nature of intestinal peristalsis. Consequently, the long-term fatigue performance and structural durability of the metamaterial under repeated loading cycles remain unexplored.

### 3.2 FE and experimental assessment of energy absorption behaviors

To evaluate the EA and SEA performance of the tubular metamaterial, the results from three experimental specimens (EXP.-1, EXP.-2, and EXP.-3) were compared with the numerical simulation data. All tests were conducted under identical geometric parameters, loading conditions, and material properties to ensure a consistent and reliable comparison.

For EXP.-1 and EXP.-2, the results closely matched the numerical predictions, with deviations within 2%, confirming the accuracy of the FE model and selected parameters. In contrast, EA and SEA decreased by 5.5% and 5.3%, respectively, for EXP.-3, primarily due to geometric imperfections introduced during 3D printing. A comparative evaluation of the energy absorption capacity and specific energy absorption is presented in Figure 8C.

### 3.3 Parametric studies

#### 3.3.1 Poisson’s ratio-strain curves under different gap configuration

**FIGURE 9:**
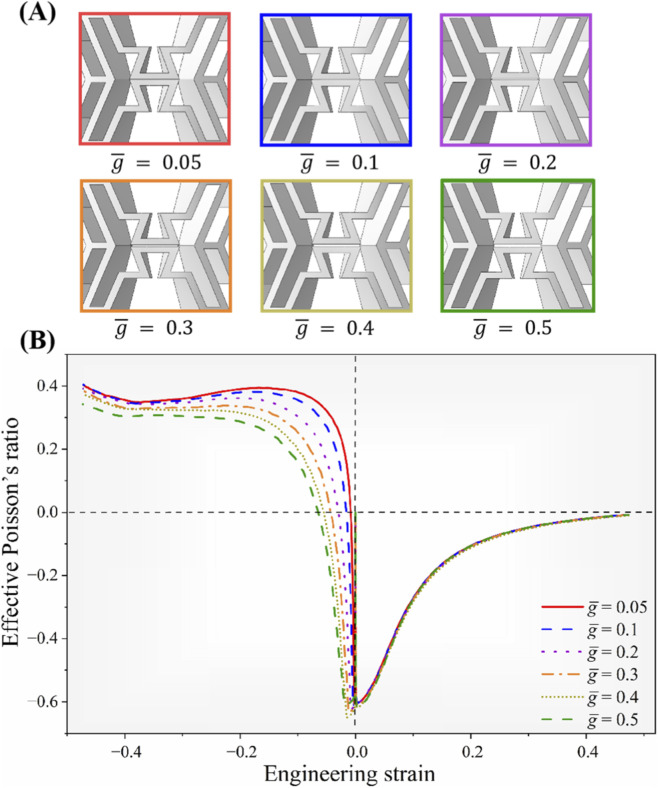
Parametric study of the effect of the gap $\overset{®}{g}$ . **(A)** Six models with different $\overset{®}{g}$ values. **(B)** Effective Poisson’s ratio-strain curves of tension and compression of the structure with six different $\overset{®}{g}$ values.

One of the key geometric parameters influencing the performance of the tubular metamaterial under compressive and tensile loading is the gap between the horizontal struts of the concave unit cells, denoted as g *.* To generalize the parametric analysis and facilitate comparison across different scales, a dimensionless parameter $\bar{g}$ ​is employed. To investigate the effect of this parameter under in-plane compression and tension, six models with different gap values ( g = 0.05 mm, 0.1 mm, 0.2 mm, 0.3 mm, 0.4 mm and 0.5 mm) were designed and analyzed using the validated FE model, as shown in Figure 9A.

Analysis of the effective Poisson’s ratio-strain curves indicates that variations in the parameter $\bar{g}$ allow the Poisson’s ratio to be effectively tuned. Under uniaxial tensile loading, the Poisson’s ratio remains nearly constant across all configurations, demonstrating stable deformation behavior regardless of the gap size. Furthermore, in all examined cases, the NPR effect was consistently observed throughout the entire tensile loading process, as shown in Figure 9B.

In contrast, under compression, the effective Poisson’s ratio exhibited a pronounced dependence on the parameter $\bar{g}$ . Simulation results revealed that the sign-switchable Poisson’s ratio observed during compression arises from the onset of self-contact between the triangular struts. Increasing the gap $\bar{g}$ enlarges the initial spacing between the horizontal struts, requiring additional deformation to close the gap during the early loading phase. This delays the activation of the concave cells and, consequently, postpones the occurrence of the sign change in the Poisson’s ratio. Notably, for gap values of $\bar{g}=0.05$ and $\bar{g}=0.1$ , the Poisson’s ratio transitions almost instantaneously under both tension and compression, indicating high sensitivity and functional responsiveness of the design. The sign-switching behavior results from a two-stage deformation process. Consequently, the concave cells undergo elastic bending in the first stage, thereby preserving their auxetic response. Following the occurrence of self-contact, the structure exhibits resistance to further transverse contraction, thereby initiating a transition to conventional deformation.

Overall, it was showed that increasing the gap $\bar{g}$ enhances the NPR effect under compression, whereas smaller gap values promote an earlier onset of the sign-switchable Poisson’s ratio. These findings demonstrate that variation in $\bar{g}$ has little influence on the tensile stiffness but enables precise tuning of the compressive stiffness by adjusting this geometric parameter.

#### 3.3.2 Stress-strain behavior and energy absorption under different gap configurations

**FIGURE 10:**
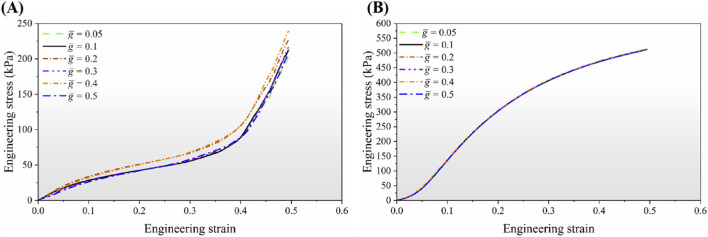
Engineering stress-strain curves of the structure with six different non-dimensional $\overset{®}{g}$ values. **(A)** Uniaxial compression loading scenario. **(B)** Uniaxial tension loading scenarios.

**FIGURE 11:**
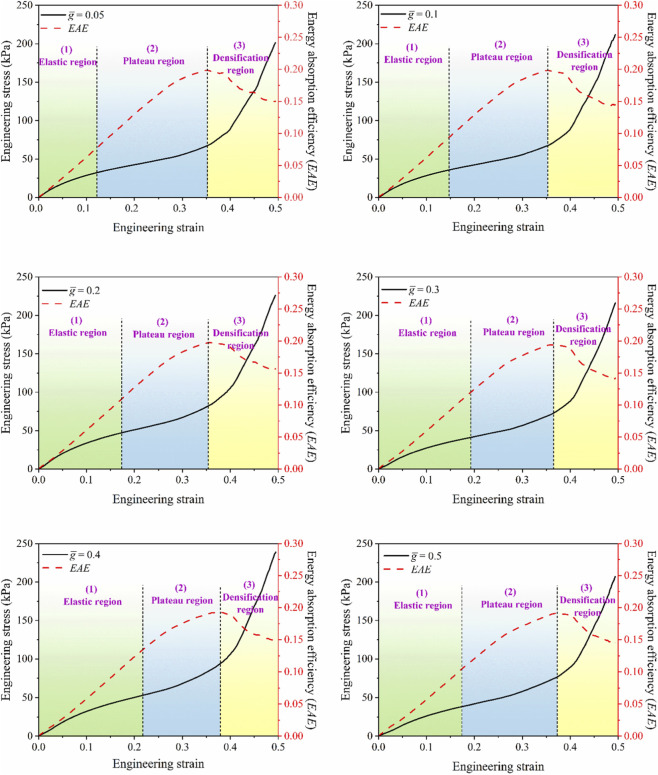
Engineering stress-strain curves of the structure under uniaxial compression loading scenario were evaluated for six distinct $\bar{g}$ values. The curves illustrate three characteristic deformation regions: (1) the elastic region, (2) the plateau region, and (3) the densification region.

**FIGURE 12:**
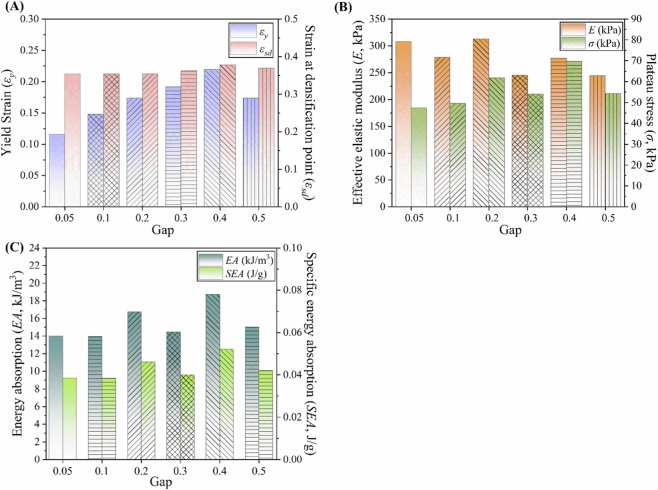
Comparison of mechanical properties at different gap sizes. **(A)** Yield strain and strain at densification point. **(B)** Effective elastic modulus and plateau stress. **(C)** Energy absorption (EA) and specific energy absorption (SEA).

The stress-strain curves obtained from the FE simulations are presented in Figure 10. The results show that the effective elastic modulus is strongly influenced by the gap parameter $\bar{g}$ , as shown in Figure 10A. As illustrated in Figure 11, each curve displays three distinct deformation regions: (1) an initial linear elastic region, (2) a plateau region with nearly constant stress, and (3) a densification region characterized by a sharp increase in stress beyond a critical strain. The compressive behavior of the metamaterial can therefore be tailored by varying $\bar{g}$ . An increase in $\bar{g}$ results in a non-monotonic trend in stiffness, as indicated by variations in E. The maximum stiffness is observed at an intermediate gap of $\bar{g}$ = 0.2, however, further increases in $\bar{g}$ reduce the structure’s resistance to elastic deformation. A partial recovery of stiffness is observed at $\bar{g}$ = 0.4. In addition, the yield strength can be independently adjusted by varying g , allowing the onset of irreversible deformation to be delayed and the structural integrity to be maintained over a wide range of loading conditions. The analysis indicates that the yield strength increases with increasing $\bar{g}$ , reaching a maximum at $\bar{g}$ = 0.4, beyond which it gradually decreases. The variations in stiffness and yield strength for structures with different $\bar{g}$ values are summarized in Figures 12A,B.

The plateau region plays a critical role in determining the energy absorption capacity of the metamaterial. The combination of a high stress plateau and a large densification strain provide a wide range of uniform compressive response before the structure transitions into the densified state. As the densification strain increases, a corresponding rise in energy absorption is observed. The gap parameter $\bar{g}$ governs the activation sequence of the deformation mechanisms. As the gap increases, the onset of self-contact between the triangular struts of the concave cells is delayed, thereby maintaining their auxetic response up to larger strains. Once contact occurs, energy absorption is enhanced through stress concentrations at the contact zones and subsequent load transfer to the convex cells, which leads to a stable plateau stress under continued deformation. The results indicate that structures with larger gaps are capable of sustaining greater deformations before entering the densification phase. The EA and SEA—key parameters for evaluating materials used in protective and damping systems—are positively tunable with increasing gap size $\bar{g}$ , as shown in Figure 12C. The maximum EA and SEA values were obtained at $\bar{g}=0.4$ , corresponding to the highest densification strain, while the minimum values were recorded at $\bar{g}=0.05$ .

In contrast, the tensile behavior response of the structure exhibits fundamentally different characteristics. As shown in Figure 10B, all configurations demonstrate a monotonically increasing stress-strain relationship without a distinct plateau region. The curves for all gap sizes are nearly identical, indicating that the geometric gap parameter has minimal influence on the tensile mechanical response. The lack of significant variation in tensile behavior across different gap configurations is attributed to the absence of self-contact mechanisms during tensile loading, which prevents the sign-switching phenomenon observed under compression.

## 4 Conclusion

In this study, a novel tubular mechanical metamaterial was developed by integrating re-entrant unit cells exhibiting a NPR with hexagonal unit cells displaying a PPR, resulting in a structure capable of sign-switchable Poisson’s ratio behavior and tunable mechanical properties. The following conclusions were drawn:

1. The design is governed by self-contact mechanisms between triangular struts, which activate under compression, enabling the Poisson’s ratio to transit from negative to positive. In contrast, the structure maintains a consistently negative Poisson’s ratio under tensile loading. This distinct deformation behavior allows for programmable mechanical responses depending on the loading mode.
2. The geometric gap between the horizontal struts of the concave unit cells was identified as a key tuning parameter. Under tensile loading, the Poisson’s ratio remains stably negative across all gap sizes, indicating that tensile behavior is largely insensitive to variations in $\bar{g}$ . Under compression, however, increasing g enlarges the initial spacing between struts, delaying the onset of self-contact and thus postponing the sign-switching transition from negative to positive. By adjusting the geometric gap, precise control over the transition strain is achieved, thereby amplifying the NPR effect during the early phase of compression. Therefore, the adaptive mechanical behavior enables the tailoring of mechanical performance for varying loading conditions in intestinal stent applications.
3. Both the effective stiffness and yield strength were found to be tunable through variations in g , with the maximum stiffness occurring at $\bar{g}=0.2$ and the peak yield strength at $\bar{g}=0.4$ . Energy absorption (EA) and specific energy absorption (SEA) also increased with larger gap sizes, reaching their maximum at $\bar{g}=0.4$ due to extended plateau regions and higher densification strains. Therefore, this adjustability enables the design of stents that optimally balance flexibility during implantation, long-term radial support, and the ability to energy absorption of peristaltic contractions.
4. Although the proposed design demonstrates promising mechanical properties, the present study is limited to quasi-static tests. To fully assess its suitability for intestinal stent applications, further investigations, including cyclic fatigue tests simulating repeated intestinal peristalsis, are necessary.

Overall, the data presented in the present study provide important information for the selection and design of tubular metamaterial for intestinal stent applications.

## Data Availability

The original contributions presented in the study are included in the article/supplementary material, further inquiries can be directed to the corresponding author.
